# Context-Dependency in the Effects of Nutrient Loading and Consumers on the Availability of Space in Marine Rocky Environments

**DOI:** 10.1371/journal.pone.0033825

**Published:** 2012-03-23

**Authors:** Fabio Bulleri, Bayden D. Russell, Sean D. Connell

**Affiliations:** 1 Southern Seas Ecology Laboratories, School of Earth and Environmental Sciences, The University of Adelaide, South Australia, Australia; 2 Dipartimento di Biologia, Università di Pisa, CoNISMa, Pisa, Italy; 3 Dipartimento di Scienze Botaniche, Ecologiche e Geologiche, Università di Sassari, Sassari, Italy; National Institute of Water & Atmospheric Research, New Zealand

## Abstract

**Background:**

Enhanced nutrient loading and depletion of consumer populations interact to alter the structure of aquatic plant communities. Nonetheless, variation between adjacent habitats in the relative strength of bottom-up (i.e. nutrients) versus top-down (i.e. grazing) forces as determinants of community structure across broad spatial scales remains unexplored. We experimentally assessed the importance of grazing pressure and nutrient availability on the development of macroalgal assemblages and the maintenance of unoccupied space in habitats differing in physical conditions (i.e. intertidal *versus* subtidal), across regions of contrasting productivity (oligotrophic coasts of South Australia *versus* the more productive coasts of Eastern Australia).

**Methodology/Principal findings:**

In Eastern Australia, grazers were effective in maintaining space free of macroalgae in both intertidal and subtidal habitats, irrespective of nutrient levels. Conversely, in South Australia, grazers could not prevent colonization of space by turf-forming macroalgae in subtidal habitats regardless of nutrients levels, yet in intertidal habitats removal of grazers reduced unoccupied space when nutrients were elevated.

**Conclusions/Significance:**

Assessing the effects of eutrophication in coastal waters requires balancing our understanding between local consumer pressure and background oceanographic conditions that affect productivity. This broader-based understanding may assist in reconciling disproportionately large local-scale variation, a characteristic of ecology, with regional scale processes that are often of greater relevance to policy making and tractability to management.

## Introduction

Understanding the context dependency of ecological observations offers a framework to establish the extent to which local studies may be representative of broader areas [1]. For example, knowledge of latitudinal gradients can reconcile seemingly discordant series of local observations from north to south because they can be related to a larger-scale pattern (e.g. consumer pressure [2]; species interaction strength [3]). Given that most ecological research is, and will continue to be done at local scales, broader scale studies will be key because they inform our interpretation of nature (patterns and processes).

In the marine environment, several studies have empirically examined patterns of distribution of species in the same habitat over broad spatial scales [4]–[8]. However, only a small number of studies have attempted to identify the mechanisms determining variation in the distribution of organisms at such broad scales (e.g. [2], [5], [9], [10]) and, to the best of our knowledge, none of these assessed generalities across different habitats. For instance, although intertidal and subtidal habitats are tightly linked by the transport of nutrients and pollutants [11], variation in the relative importance of bottom-up (e.g. nutrients) *versus* top-down (e.g. grazing) forces as determinants of the structure of intertidal and subtidal assemblages across broad spatial scales remains unexplored.

The effects of enhanced nutrient loading and depletion of natural populations of consumers have been shown to interact to alter diversity, evenness and biomass of plant assemblages [12]–[16]. In aquatic environments, grazing by herbivores can counterbalance positive effects of enhanced levels of nutrients on growth of primary producers [17]–[20]. Thus, decreased grazing pressure and enhanced nutrient inputs have been identified as the main determinants of the domination of coral reefs [21] and rocky habitats [22] by opportunistic macroalgae.

Recently, Burkepile and Hay [23] used a meta-analysis approach to synthesize results from 50 small scale studies reporting on the effects of the manipulation of nutrients and herbivores on a variety of intertidal or subtidal primary producers (micro- and macroalgae, seagrass and marsh plants). This synthesis suggests that the relative importance of top-down versus bottom-up forces varies among different functional groups of algae and according to background productivity levels. Further evidence of this context-dependency in the role of consumers and nutrients in structuring benthic communities has been provided by experiments investigating algae-herbivore interactions among regions characterized by different oceanographic conditions [9], [24], [25]. No empirical study has, however, assessed how the response of primary producers to alterations in nutrient inputs and grazing pressure can vary between contiguous habitats in high- *versus* low-productivity systems.

In rocky benthic habitats, the provision of free space by disturbance is crucial for the recruitment and persistence of many invertebrates and macroalgae [26]. The relative strength of top-down and bottom-up forces determines the speed at which space is re-occupied after disturbances; recovery of macroalgal assemblages is reportedly slower when grazing pressure is high, yet fostered by enhanced nutrients supply [27], [28]. Variation in the availability of free space therefore provides an estimate of the outcome of the interplay between bottom-up and top-down forces that is not biased by differences in life-history traits among species thriving in different habitats.

This study aims to fill in the gap in empirical studies by experimentally investigating how the relative importance of top-down (i.e. grazing by gastropods) *versus* bottom-up forces (i.e. nutrient loading) on patterns of space occupancy varies between rocky habitats (i.e. intertidal *versus* subtidal) characterized by fundamentally different physical conditions. In particular, the main aim was that of assessing the relative importance of grazing pressure and nutrient availability on the maintenance of unoccupied space in distinct habitats, across regions differing in intrinsic productivity (oligotrophic water of South Australia *versus* nutrient richer waters of Eastern Australia).

## Methods

### Study sites

This study was carried out on wave-exposed coasts of New South Wales and South Australia (hereafter referred to as EA and SA). All necessary permits were obtained (South Australian Fisheries exemption #98/0917 and New South Wales Scientific Collection Permit #P05/0137-2.1). Two current systems with differing nutrient regimes dominate Australia's temperate coast: the East Australian Current flowing down eastern Australia and the Leeuwin Current flowing down Western Australia and towards South Australia. The Leeuwin Current has a lower nutrient status than the East Australian Current [29], which is observed in lower chlorophyll *a* concentrations (a proxy for nutrient concentration) in South Australia than eastern Australia (e.g. 0.31–0.41 µg L^−1^ and 0.32–1.35 µg L^−1^, respectively; [30]) and *in situ* nitrogen concentrations in South Australia [30]–[32]. Thus, SA waters can be regarded as more oligotrophic than the more nutrient rich EA waters.

**Figure 1:**
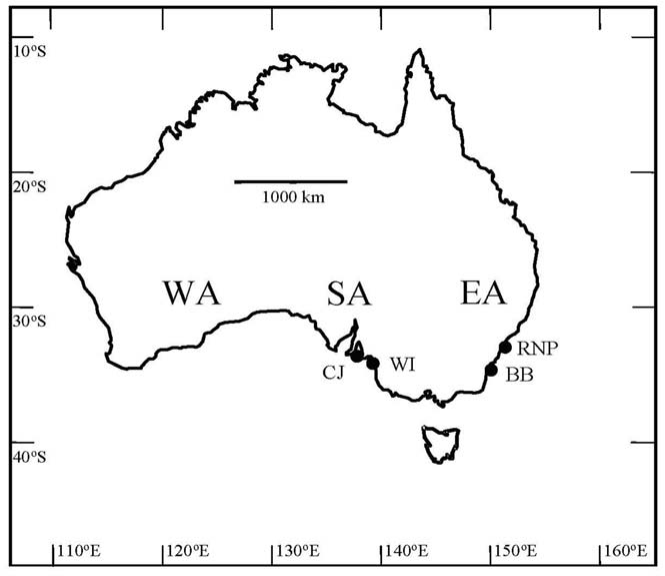
Map showing study sites on the coasts of Eastern Australia (EA) and South Australia (SA). CJ = Cape Jarvis; WI = West Island; BB = Batemans Bay; RNP = Royal National Park.

In order to evaluate variation in the development of fouling assemblages within each region, two locations (Fig. 1) were randomly chosen in both EA (Royal National Park = RNP; Batemans Bay = BB) and SA (West Island = WI; Cape Jervis = CJ).

In EA, low shore intertidal assemblages are generally characterized by the dominance of a variety of foliose, coarsely branched and filamentous algae and by the lack of grazers [33], [34]. Grazers have been, in fact, shown to be unable to thrive within dense algal beds occurring at low-shore levels [34]. Above this algal band, apart for the presence of *Hormosira banksii* and *Corallina officinalis* in pools, space is generally monopolized by the red encrusting alga, *Hildenbrandia rubra* [33]. A great variety of grazing gastropods, including the snails *Nerita atramentosa*, *Bembicium nanum* and *Austrocochlea porcata* and the limpets *Cellana tramoserica*, *Patelloida* ssp. and *Siphonaria denticulata*, is commonly found at this height on the shore. Detailed description of intertidal macroalgal and invertebrate assemblages in EA can be found elsewhere [33], [35].

As in EA, mid-shore rocky intertidal assemblages in SA have striking vertical patterns, ranging from almost devoid of algae in the upper shore zones, to being dominated by erect turf-forming, foliose and other macroalgae in the low shore zone. Grazers are almost absent within the dense zone of erect macroalgae dominating space at low-shore levels, while gastropods, such as the limpet *Cellana solida*, the chiton *Plaxiphora albida* and the gastropods *Bembicium* spp. and *Nerita atramentosa*, are common at upper levels on the shore. Both in EA and SA, experiments were done in the zone just above the low shore band of foliose macroalgae, hosting diverse grazer assemblages.

As in many other temperate regions worldwide, shallow subtidal rocky reefs in EA are characterized by stands of canopy-forming algae, mainly composed of the kelp, *Ecklonia radiata*, alternating with barren grounds dominated by encrusting coralline algae that are produced by the foraging activity of the black sea urchin, *Centrostephanus rodgersii* [1], [36]. *E. radiata* provides suitable habitats for a diverse understorey assemblage, including encrusting algae, articulated coralline algae, sponges, ascidians and bryozoans. Within kelp stands, *C. rodgersii* is uncommon, but grazing gastropods, such as the snails *Turbo torquata* and *Australium tentoriformis*, can be locally abundant.

In contrast to EA, subtidal assemblages on shallow subtidal rocky reefs in SA are characterized by the lack of barren grounds dominated by encrusting corallines, most likely as a consequence of the absence of large herbivores, such as fish or sea urchins [1]. The purple sea urchin, *Heliocidaris erythrogramma*, is present on these reefs, but, being a drifter-feeder, has weak effects on benthic algal assemblages [37]. Grazing gastropods, such as *Clanculus* spp., *Turbo* spp., *Astralium aureum*, *Granata imbricata* and *Phasianella* spp. are common within mixed stands of canopy-forming algae, including *E. radiata*, *Cystophora* spp. and *Scytothalia* spp. [38]. In order to enhance comparability between regions, subtidal experiments in both EA and SA were carried out within small clearances in kelp stands.

### Experimental design

**Figure 2:**
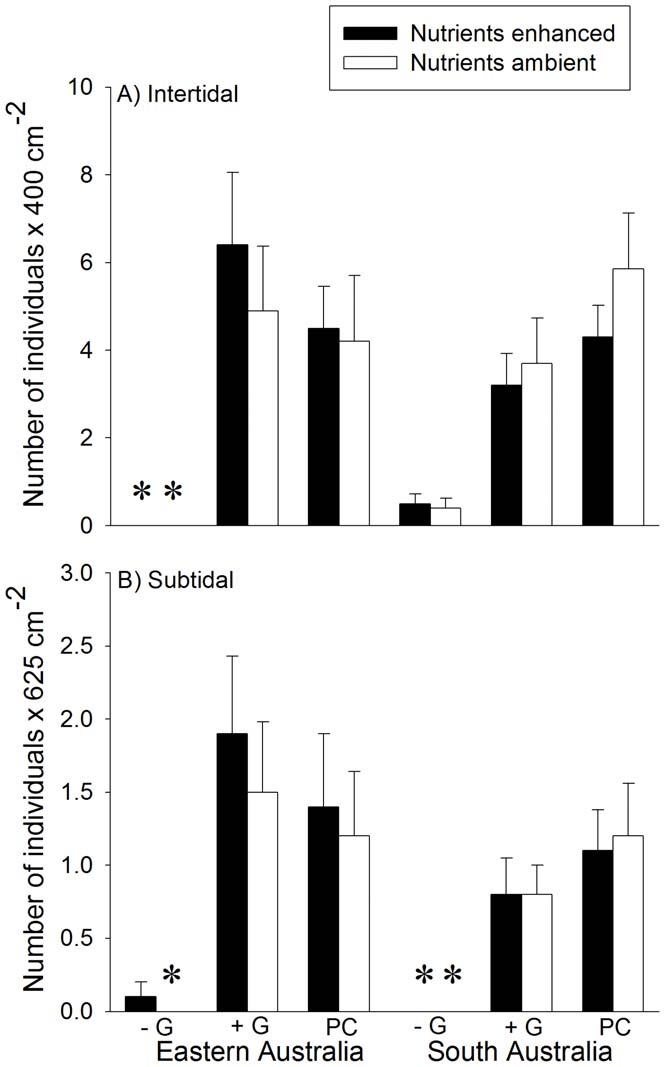
Grazer density in intertidal and subtidal habitats. Mean density (±1 SE) of gastropod grazers in A) intertidal and B) subtidal habitats in Eastern and South Australia, across the duration of the experiment; values are averages across locations (*n* = 10). Asterisks indicate values equal to zero. +G = Grazers present; −G = Grazers excluded; PC = Procedural control for the use of fences.

The experiment was set up in early November 2005 and experimental conditions were maintained throughout the austral summer. Grazers and nutrients were manipulated in both intertidal and subtidal habitats at each of the two locations within each region. At each study location, thirty 20×20 cm quadrats were randomly marked, 10 s of cm apart, on rocky platforms, between 0.5 and 0.7 m above the mean low tide and, on shallow rocky reefs, at a depth ranging from 5 to 8 m. Five quadrats were then randomly assigned to each of the six combinations of grazer (3 levels: grazers present = +G, grazers removed = −G and procedural control due to fencing = PC) and nutrient (2 levels: ambient and elevated) treatments. Grazers were excluded from −G plots by means of fences. In the intertidal, fences were made of plastic mesh (1 cm×1 cm mesh size) reinforced with a 0.5 cm×0.5 cm galvanized iron mesh and were 20×20 cm side, 4 cm high and with an outurned lip 1 cm wide. The use of mesh of two sizes was necessary to ensure exclusion of grazers (fine mesh) and resistance to breaking waves (coarse mesh). Fences were anchored to the substratum by means of stainless steel screws inserted in rawl plugs and epoxy putty was applied at corners to obtain an effective seal. Partial fences (2 sides), allowing grazers to move in and out of experimental plots, were used to control for potential artifact effects generated by grazer exclusion devices. In the subtidal, fences, made of an external 2 cm×2 cm and by an internal 0.5 cm×0.5 cm galvanized iron mesh layer, were 25 cm a side, 22 cm high, with an outurned lip 3 cm wide. These fences were tied with plastic cable-ties to 16 mm diameter×42 mm long bolts cemented with epoxy putty into holes drilled in the rock. Grazing by herbivorous fish is weak within *E. radiata* stands [39] and there was, therefore, no need to apply a roof to fences. Damaged fences were replaced and the efficacy of the treatments was checked at roughly 1-month intervals. Fencing was effective in excluding grazers from experimental plots across regions and habitats (Fig. 2). Few individuals were found inside fences during visits in the field and these were generally small in size. Densities of grazers were comparable between open and partial fences controlling for procedural artefacts (Fig. 2).

In order to standardize the nature of the substratum among locations and habitats, the development of algal assemblages was assessed on 12 cm×12 cm, fibre-cement plates (Hardi-flex, 4.5 mm in thickness) which were fixed to the substratum, in the centre of plots, by means of stainless steel screws and cable-ties in the intertidal and subtidal, respectively.

Nutrient levels were elevated by using Osmocote® fertilizer pellets (6 mo release: 17, 4.3, 8.2 N-P-K). This approach provides a realistic and gradual nutrient release and has been indicated as the most appropriate method of elevating nutrients in subtidal experiments [40]. It has been successfully applied in previous experiments performed at our study sites [30], [32], [41] and elsewhere [42]–[44]. It is, however, worth noting that in field experiments nutrient levels are not fixed (i.e. they vary according to ambient variation), but elevation is fixed (i.e. ambient *versus* elevated).

Nutrient enrichment of the water column was achieved through the deployment of two 20 cm long bags made of nylon shading cloth (1-mm mesh size) and containing 80 g of fertiliser, at a distance of about 5 cm from experimental plates. These bags were fixed with cable ties on the internal side of the cages, or, in the case of open plots, by means of stainless steel screws inserted into rawl plugs. This method has been used to assess the effects of elevated nutrients [40], [41] and previous studies have not detected artefacts associated with the physical presence of mesh bags containing spherical balls of Osmocote® pellets [32].

Nutrient bags were replaced monthly, ensuring the maintenance of experimental conditions [30]. Water samples were also taken in the proximity of subtidal plates 12 wks after the start of the experiment (about 1 mo after the last nutrient bag replacement and before the deployment of new bags) to assess whether nitrogen concentrations had indeed been increased in experimental plots of elevated nutrients and whether ambient nitrogen was greater in eastern than southern Australia. Two water samples were taken approximately 3 cm above the centre of each plate, using a 25 ml syringe. Samples were shaken and filtered with a 0.45 µm filter and frozen for transport to the laboratory for analysis. Nitrate concentrations (mg l^−1^) indicate that elevated nutrient levels were achieved across all sites within EA (RNP: ambient = 0.157±0.07; elevated = 0.272±0.086; BB: ambient = 0.017±0.003; elevated = 0.938±0.13; data are mean ± SE values averaged across open and fenced plots within each site; *n* = 10) and SA (CJ: ambient below detection limits of 0.001; elevated = 1.531±0.10; WI: ambient = 0.004±0.000; elevated = 0.04±0.012; *n* = 10). Relative to nitrogen, phosphorous is not generally considered to be a limiting nutrient in near shore coastal waters (but see [45]). Enrichments in phosphorus can be difficult to detect following the filtering of water samples, which removes most of the ionic phosphorus [30]. Thus, it was not assessed in our estimates of nutrient concentrations.

Settlement plates were sampled 17 weeks after the experiment was started. Percent cover of sessile organisms was estimated *in situ* using the point-intersect method as applied to a grid of 25 evenly spaced points over the central 10×10 cm of the each plate.

The cover of encrusting corallines, biofilm (a thin layer of blue-green algae) and bare space were combined into a single category, in order to test the hypothesis that different combinations of grazer and nutrient treatments would determine a different amount of primary space. Encrusting corallines are, in fact, weak competitors and represent a suitable substratum for the recruitment of a number of macroalgae and sessile invertebrates. The use of this variable, hereafter referred to as unoccupied space, enables comparisons between habitats and regions unbiased by variation in life-history traits of colonizers between habitats or regions. However, the response of algal turfs (composed of filamentous species) and foliose algae (mostly composed of *Ulva* spp., *Enteromoprha intestinalis* and *Porphyra umbilicalis*) was examined to enhance the interpretation of variations in free space in response to experimental conditions.

Data were analyzed by means of a five-factor ANOVA, including: (1) Habitat (2 levels, fixed and crossed with Region, Grazers and Nutrients); (2) Region (2 levels, fixed and crossed with Habitat, Grazers and Nutrients); (3) Location (2 levels, random, nested into Region and crossed with Habitat, Grazers and Nutrients); (4) Grazers (3 levels, fixed and crossed with all the other factors); (5) Nutrients (2 levels, fixed and crossed with all the other factors). Homogeneity of variances, checked by means of Cochran's test, could not be achieved by transformation for unoccupied space, but data were analyzed nonetheless since analysis of variance is robust for departure from this assumption when there are many independent replicates and sizes of samples are equal [46]. SNK tests were used for *a posteriori* comparison of the means [46].

## Results

**Figure 3:**
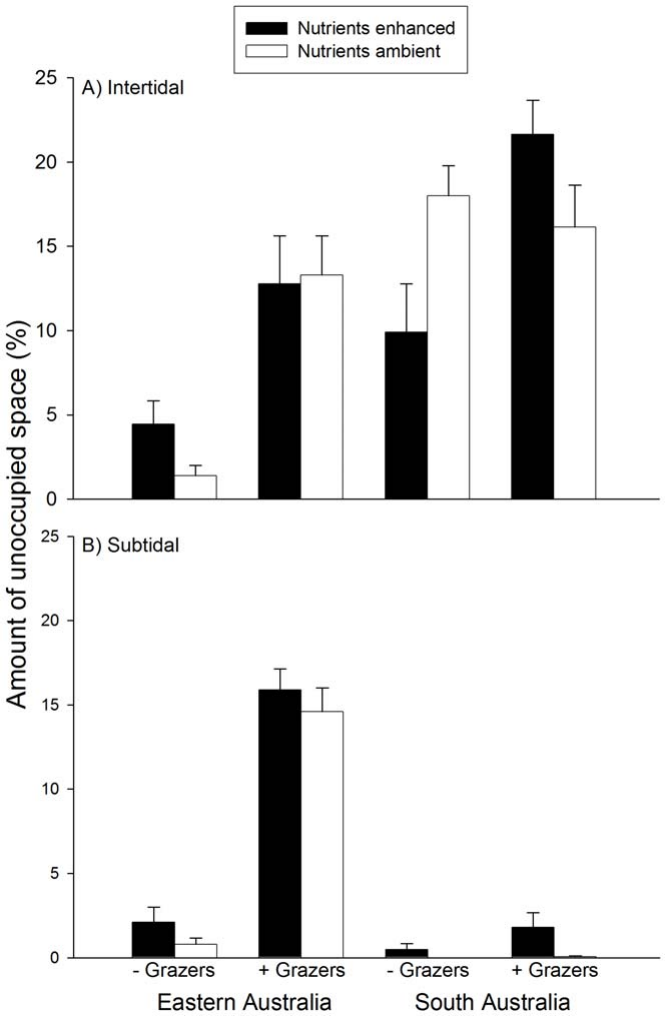
Amount of unoccupied space (mean % ±1 SE) for different combinations of grazer and nutrient treatments. A) intertidal and B) subtidal habitats in Eastern and South Australia, 17 weeks after the start of the experiment. Values are averages across locations (*n* = 10).

The amount of unoccupied space was influenced by the manipulation of grazers and nutrients, but not consistently between habitats and regions (Fig. 3, Table 1). In intertidal habitats, the removal of grazers reduced the availability of unoccupied space regardless of nutrient levels in EA, whilst such an effect was recorded in SA only at enhanced nutrient levels (Fig. 3A, SNK tests). Removing grazers from subtidal habitats in EA had negative effects on the availability of unoccupied space that were independent of nutrient levels and greater than those recorded in intertidal habitats (Fig. 3B, SNK tests). In contrast, no difference among grazer treatments emerged in subtidal habitats of SA (Fig. 3B, SNK tests).

**Table 1:** ANOVAs on the effects of Habitat (Intertidal vs Subtidal), Region (EA vs SA), Location, Grazers (Present, Removed and Procedural control) and Nutrients (Enhanced vs Ambient) on the amount of unoccupied space (%), percentage cover of algal turfs and foliose macroalgae, 17 weeks after the experiment was started.

		Unoccupied space		Algal turfs		Foliose macroalgae	
Source of variation	df	MS	*F*	MS	*F*	MS	*F*
Habitat = H	1	4657.41	149.98***	78940.45	52.90	821.40	1.27
Region = R	1	36.62	0.20	28181.11	3.61	2522.02	3.91
Location (R) = L(R)	2	186.56^a^	8.94***	7806.82	19.35***	645.41	4.36*
Grazers = G	2	1635.98	35.71**	15789.64	18.04*	3469.76	22.72**
Nutrients = N	1	12.72	0.16	254.89	0.25	308.27	1.14
H×R	1	4710.42	151.68**	44154.98	29.59*	1392.02	2.15
H×L(R)	2	31.05^a^	1.49	**1492.25**	3.70*	**647.04**	4.37*
H×G	2	82.59	1.08	1190.89	1.58	67.76	0.61
H×N	1	26.83	0.43	222.98	0.78	504.60	5.45
R×G	2	489.84	10.69*	2672.99	3.05	1182.88	7.75*
R×N	1	4.61	0.06	1341.11	1.30	43.35	0.16
G×L(R)	4	45.82^a^	2.20	875.06	2.17	152.72	1.03
N×L(R)	2	81.31^a^	3.90*	1033.38	2.56	270.01	1.82
G×N	2	90.85	1.57	1540.87	1.92	**690.40** **^c^**	4.66*
H×R×G	2	115.50	1.51	446.37	0.59	**2383.88**	21.33**
H×R×N	1	33.94	0.55	363.42	1.27	74.82	0.81
G×H×L(R)	4	**76.33** a	3.66**	754.07	1.87	111.75	0.75
H×L(R)×N	2	62.02^a^	2.97	285.21	0.71	92.64	0.63
H×G×N	2	29.85^a^	1.43	467.18	1.16	270.24	1.12
R×G×N	2	112.78	1.96	2356.81	2.93	18.24	0.12
N×G×L(R)	4	**57.68** a	2.76*	804.28	1.99	138.12‡	eliminated
H×R×G×N	2	**90.97** a	4.36*	**2166.67** b	5.37**	11.00	0.05
H×L(R)×G×N	4	18.96‡	pooled	569.39†	eliminated	240.35	1.62
Residual	192	20.91		403.52		148.02	

Pooling procedures were used according to Underwood (1997). * = *P*<0.05; ** = *P*<0.01, *** = *P*<0.001. Higher order interactions relevant for testing proposed hypotheses are reported in bold.

a Tested against the pooled term: Residual+H×L(R)×G×N (df = 196; MS = 20.87).

b, c Tested against the Residual;

‡ not significant at *P* = 0.25;

† not significant at *P* = 0.23.

**Figure 4:**
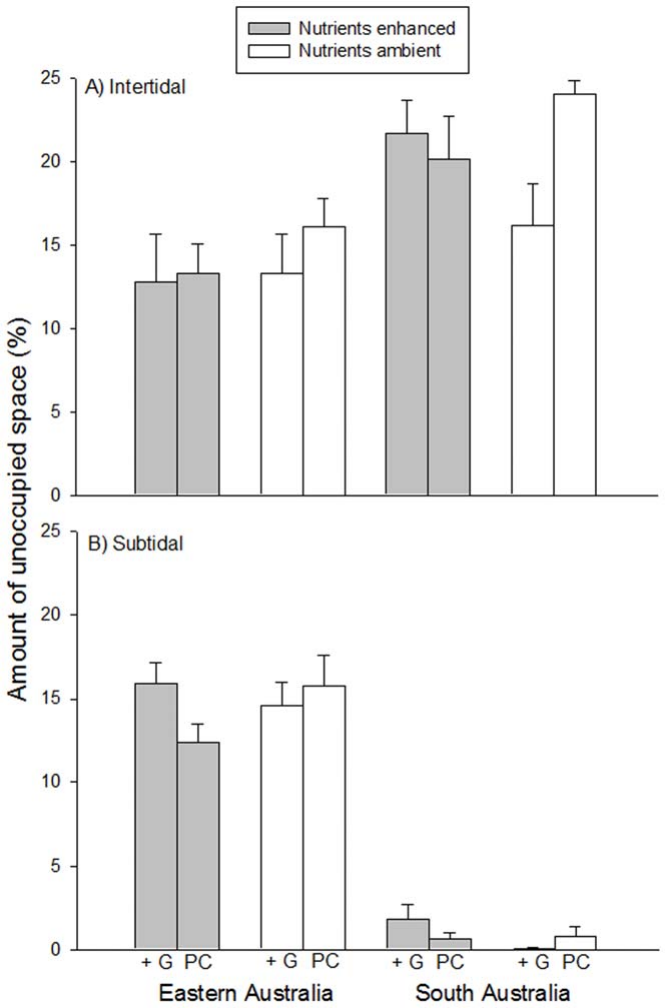
Amount of unoccupied space (mean % ±1 SE) in open and procedural control plots for the different combinations of region, habitat and nutrient levels. Grazers present = +G; Procedural control = PC; (A) intertidal and (B) subtidal habitats. Values are averages across locations (*n* = 10).

The effects of grazers on the amount of unoccupied space also varied at smaller scale, that is, between locations within each region (Table 1). These effects were complex, as variability from one location to another was not consistent between habitats (i.e. significant Grazers×Habitat×Location (Region) interaction) and was influenced by the manipulation of nutrients (i.e. significant Nutrient×Grazers×Location (Region) interaction). However, *a posteriori* comparisons indicated inconsistencies in the magnitude of effects between top-down versus bottom-up forces among locations in a subset of levels of some factors (Table 2). In these cases, variation between locations was often due to the magnitude of differences between open and procedural control plots at one of the two locations (Table 2). In contrast, when compared at the regional level, the amount of unoccupied space was significantly greater in procedural controls than open plots only in South Australian intertidal habitats, at natural nutrient levels (Fig. 4A). Given that densities of grazers did not differ much between procedural controls and open treatments (Fig. 2A), it could be argued that grazing intensity was greater in procedural controls than in open plots.

**Table 2:** *A posteriori* comparisons for unoccupied space.

Nutrients×Grazers×Location(Region)		
**EA**	**Royal National Park**	**Batemans Bay**
	Nutrients elevated: PC = +G>−G	Nutrients elevated: +G>PC>−G
	Nutrients ambient: PC = +G>−G	Nutrients ambient: PC = +G>−G
**SA**	**West Island**	**Cape Jervis**
	Nutrients elevated: +G = PC>−G	Nutrients elevated: +G = PC = −G
	Nutrients ambient: PC = −G = +G	Nutrients ambient: PC = +G = −G

Grazers×Habitat×Location(Region)		
**EA**	**Royal National Park**	**Batemans Bay**
	Intertidal: PC>+G>−G	Intertidal: +G = PC>−G
	Subtidal: +G = PC>−G	Subtidal: +G = PC>−G
**SA**	**West Island**	**Cape Jervis**
	Intertidal: PC>+G>−G	Intertidal: +G = PC>−G
	Subtidal: +G = PC = −G	Subtidal: −G = PC = +G

SNK tests for higher-order interactions including the factors Grazers, Nutrients and Location; −G = −Grazers; +G = +Grazers; PC = Procedural control.

**Figure 5:**
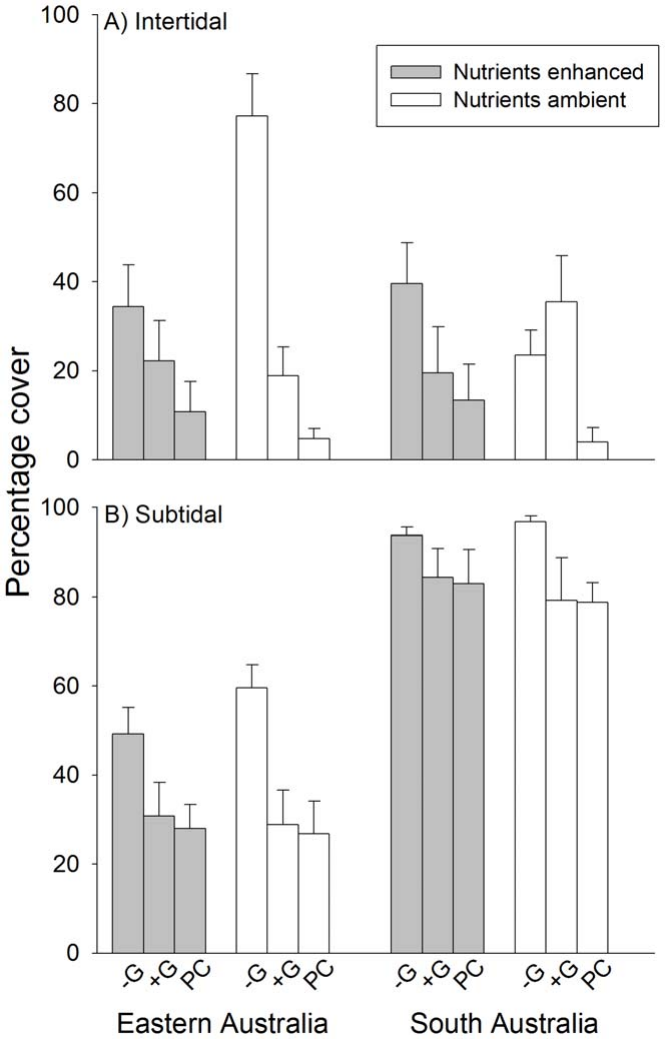
Percentage cover (mean ±1 SE) of algal turfs for different combinations of grazer treatments and nutrient levels in Eastern and South Australia. Grazer removal = −G; Grazers present = +G; Procedural control = PC; (A) intertidal and (B) subtidal habitats. Values are averages across locations (*n* = 10).

The removal of grazers had significant effects on the cover of algal turfs that varied between habitats, regions and nutrient levels (Table 1, Fig. 5). In EA, the exclusions of grazers promoted the development of algal turfs in both intertidal and subtidal habitats, but only where nutrients were left at ambient levels (Fig. 5A–B, SNK tests). In the intertidal of SA, the removal of grazers resulted in an enhancement of the cover of algal turfs only at elevated nutrient levels (Fig. 5A). At ambient nutrient levels, the cover of these algal forms did not differ between control and grazer removal plots, while it was very small in procedural controls. This suggests that the presence of fences might have enhanced grazing rates, probably by providing shelter from adverse environmental conditions (e.g. desiccation) (Fig. 5A). In the subtidal, algal turfs were not affected by grazers and, by the end of the experiment, monopolized space on fouling plates, irrespective of nutrient levels (Fig. 5B, SNK tests).

In addition, variations in the cover of algal turfs between habitats varied from one location to the other (Table 1). Except for one of the locations in EA (BB), the cover of turfs was significantly greater in subtidal than intertidal habitats (SNK tests).

**Figure 6:**
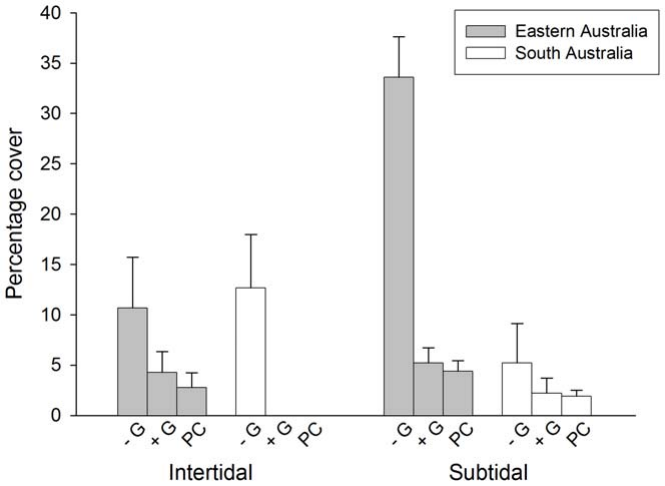
Percentage cover (mean ±1 SE) of foliose macroalgae for different grazer treatments in intertidal and subtidal habitats of Eastern and South Australia. Grazer removal = −G; Grazers present = +G; Procedural control = PC.; values are averages across locations and nutrient levels (*n* = 20).

**Figure 7:**
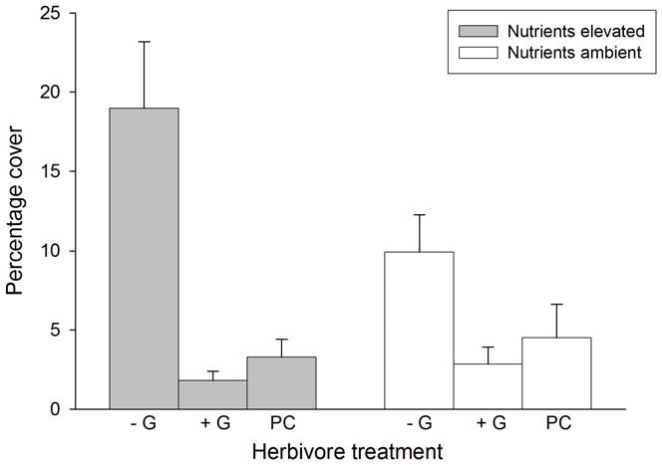
Percentage cover (mean ±1 SE) of foliose macroalgae for different combinations of grazer treatment and nutrient levels. Grazer removal = −G; Grazers present = +G; Procedural control = PC. Values are averages across habitats and regions (*n* = 40).

There were interactive effects of grazers and nutrients on foliose macroalgae that varied between regions (Table 1). The removal of grazers resulted in a significant increase in the cover of these algal forms in subtidal habitats of EA and in intertidal habitats of SA (Fig. 6, SNK tests). Although not significant, a similar trend was evident also for intertidal shores of EA. The analysis also showed significant interactive effects of grazers and nutrients on foliose macroalgae that was consistent between habitats and regions (Table 1). The removal of grazers enhanced the cover these algal forms at both natural and elevated nutrient levels (Fig. 7, SNK tests). However, the positive effect of grazer removal was greater when nutrient levels were elevated (Fig. 7, SNK tests). Finally, differences between habitats in the cover of foliose macroalgae were not consistent between locations within regions (Table 1). There were no significant differences between habitats at locations in SA, while covers were significantly greater in the subtidal than the intertidal at one of the locations in EA (RNP; SNK tests).

## Discussion

**Figure 8:**
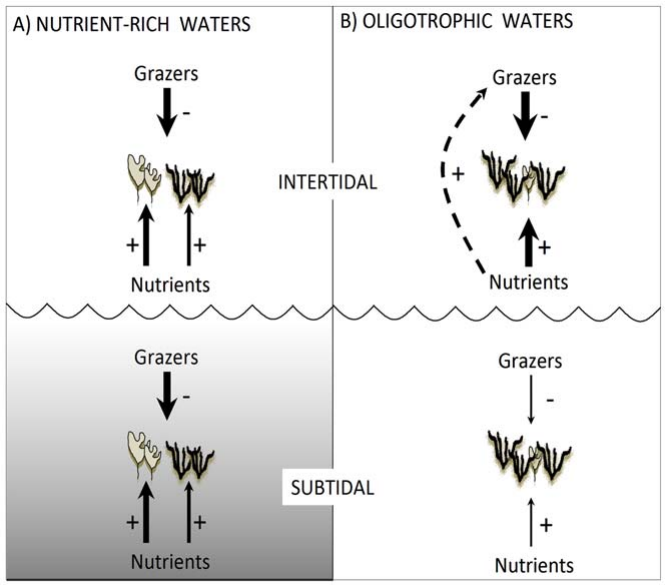
Diagram showing the relative role of nutrients (bottom-up) and grazers (top-down) in intertidal and subtidal habitats in (A) nutrient-rich and (B) oligotrophic waters. Arrows show direct (solid line) and indirect (dashed line), positive (+) and negative (−) effects. The thickness of the arrows reflects their strength. Black, bushy drawings represent algal turfs, grey leafy drawings represent foliose macroalgae.

The relative importance of grazers and nutrients in controlling the development of ephemeral macroalgae (i.e. turf-forming and foliose forms) and, hence, in maintaining unoccupied space was strongly context-dependent, varying between habitats and according to background environmental conditions (i.e. productivity). In Eastern Australia, grazers were largely effective in maintaining space free of macroalgae in both intertidal and subtidal habitats, irrespective of natural or enhanced levels of nutrients, suggesting a prevalence of top-down forces (Fig. 8A). In contrast, in South Australia, grazers could not prevent colonization of space by macroalgae in subtidal habitats regardless of nutrient levels, yet, in intertidal habitats, they were effective in maintaining unoccupied space where nutrients were elevated (Fig. 8B). These findings, while adding to the growing body of evidence that background productivity regulates interactive effects of consumers and nutrients on primary producer assemblages [14], [24], [25], [47], show that, within a regional context, the relative strength of these forces can vary across habitats.

These regional patterns in the relative importance of top-down versus bottom-up forces between habitats emerged despite large variability at the scale of 10 s of km (i.e. between locations within regions). Variation in the structure and development of rocky assemblages at this scale has been previously documented both in intertidal and subtidal habitats along temperate coasts of Australia [35], [48], and elsewhere (New Zealand [49]; Mediterranean [50]; North-east Pacific [51]). It is worth recognizing that measures of processes that maintain unoccupied space enable direct comparisons among contrasting habitats of distinct taxonomies and life-histories, thereby assisting the identification of general patterns and responses in nature.

Densities of grazing gastropods were generally greater in intertidal than subtidal habitats, but varied less between regions. Thus, variations in grazer density alone may not explain variation in their effects at a regional scale. Variations in the species composition of herbivore assemblages might have contributed to differences in their grazing effects between regions. Unfortunately, detailed information on grazing efficiency is not available for some of the gastropod species present (in particular, for SA), making it difficult to speculate over the role of species-specific traits in determining their ability to control macroalgal development.

In EA, increased development of both turf-forming and foliose macroalgae following the removal of grazers indicates a prevalence of top-down control that was consistent across habitats and was not affected by enhanced nutrient loading, similar to previous studies in both intertidal [33] and subtidal rocky habitats [36]. Our study reveals the biogeographic context of this knowledge, showing that these processes are not as strong in South Australia, where both rates of productivity and consumption are regarded to be weak [1]. Our study also reveals the generality of the strength of herbivory in Eastern Australia, showing that in subtidal rocky habitats, strong herbivory is not limited to kelp-barren dynamics, but also extends to the interior of forested areas. In the relatively nutrient-rich waters of Eastern Australia, therefore, it appears that different guilds of grazers are effective in controlling primary producers across a range of rocky subtidal habitats (i.e. urchins in encrusting coralline barrens *versus* gastropods inside kelp forests).

In South Australia, where rates of coastal productivity and consumption are lower, the strength and responses of herbivory to subtidal and intertidal treatments differed. At ambient nutrient levels, removing grazers did not lead to the proliferation of turf-forming or foliose macroalgae and, as a consequence, had no effect on the amount of unoccupied space available in intertidal habitats. However, when nutrients were enhanced, removing grazers strongly promoted colonization by algal turfs, stressing the importance of bottom-up effects of nutrients in this nutrient-poor system (Fig. 8B, upper panel). In contrast, although there was a tendency for a smaller amount of unoccupied space in the absence of grazers, there was no significant effect of the experimental conditions on the availability of unoccupied space on SA subtidal rocky reefs, as a consequence of the monopolization of fouling plates by algal turfs (Fig. 8B, lower panel). Weak effects of grazing within subtidal kelp forests in SA may be due to the relatively sparse densities of grazers and difference in feeding efficiency of grazers compared to EA. Given that herbivore densities were only marginally smaller in the south than east coast, we consider that differences in grazing efficiency might have contributed to generate regional patterns.

Although nutrient levels in both EA and SA are considerably lower than those reported for other regions (i.e. Baltic [13], [27], New Zealand [24], NE Pacific [52], NW Atlantic [27]), our patterns provide an alternative perspective from that which suggests stronger consumer control of primary producers in relatively nutrient-poor environments [14], [23]. The larger negative effect of grazers on algal turfs at elevated than ambient nutrient levels on SA intertidal rocky shores clearly shows that inputs of nutrients can directly increase rates of algal consumption by intertidal grazers under oligotrophic conditions. Thus, direct positive effects of nutrient inputs on plant productivity can be offset by indirect negative effects generated by the stimulation of grazing activity (Fig. 8B, upper panel). Nutrient enrichment can enhance the nutritional value of macroalgae [18], [53]. Indeed, fish [54] or mollusc herbivores often remove greater biomass of plant matter that has been exposed to elevated nutrients [55], [56], including the South Australian coast [19]. Increased consumption may occur as a consequence of an increase in the attraction of consumers to prey or an increase in *per capita* consumption [19], [57]. Since there was not an increase in grazer densities at elevated nutrient levels, grazing pressure on settlement plates was most likely to have increased through greater *per capita* consumption. However, it is worth noting that while this increase in the grazing by individuals has been noted in multiple cases (e.g. [19], [57]), caution may be warranted in scaling-up this effect from the size of our plots to that of whole coasts.

In the more nutrient rich waters of EA, consumption of algal turfs by herbivores was not fostered by nutrient elevation, suggesting that alterations to the N content of macroalgae generated by nutrient releases could be smaller when ambient concentrations are naturally greater and not sufficient to trigger switches in feeding rates of consumers. Rather, in EA, elevating nutrient levels resulted in a reduced development of algal turfs in the absence of grazers both in intertidal and subtidal habitats. This pattern could be indirectly generated by a stronger response of foliose species such as *Ulva* and *Enteromorpha* to nutrient inputs. A recent study, performed on intertidal rocky shores of South Island of New Zealand, has shown that, when herbivory was reduced to very low levels, enrichment generated increases in the abundance and biomass of foliose algae [58]. Although turf-forming algae have been widely shown to benefit from increased nutrient levels [32], [59], [60], our results and those of Guerry et al. [58] suggest that foliose macroalgae, in virtue of their great N-affinity, might exhibit a strong response to nutrient inputs, limiting, to some extent, the proliferation of turf-forming species (Fig. 8A).

Regardless of the mechanisms operating, the response of benthic primary producers to the removal of consumers and increase in nutrients was consistent between habitats on the east but not south coast. Grazers reduced the effects of enhanced nutrients at natural densities, suggesting that they may provide an important process in buffering nutrient inputs from human land-based activities. On the other hand, the loss or decrease of gastropod grazers from SA kelp forests would have little impact on the ability of the system to resist nutrient loading, as already observed through recent coastal urbanisation [11]. Thus, bearing in mind the uncertainties in scaling up from small experimental units to realistic eutrophication scenarios, our results warn against extending management strategies from one region to another and from one habitat to another, assuming equal influence of bottom-up versus top-down forces. Forecasting the effects of eutrophication in coastal waters requires balanced understanding between life-history traits of local guilds of consumers and background oceanographic conditions that affect productivity. This broader-based understanding may assist in reconciling disproportionately large local-scale variation, a characteristic of ecology, with regional scale processes that are often of greater relevance to policy making and tractability to management.
